# Visual Speech Perception Cues Constrain Patterns of Articulatory Variation and Sound Change

**DOI:** 10.3389/fpsyg.2018.00728

**Published:** 2018-05-15

**Authors:** Jonathan Havenhill, Youngah Do

**Affiliations:** ^1^Department of Linguistics, Georgetown University, Washington, DC, United States; ^2^Department of Linguistics, University of Hong Kong, Hong Kong, Hong Kong

**Keywords:** audiovisual speech perception, sound change, articulatory variation, ultrasound tongue imaging, misperception, Northern Cities Vowel Shift

## Abstract

What are the factors that contribute to (or inhibit) diachronic sound change? While acoustically motivated sound changes are well-documented, research on the articulatory and audiovisual-perceptual aspects of sound change is limited. This paper investigates the interaction of articulatory variation and audiovisual speech perception in the Northern Cities Vowel Shift (NCVS), a pattern of sound change observed in the Great Lakes region of the United States. We focus specifically on the maintenance of the contrast between the vowels /ɑ/ and /ɔ/, both of which are fronted as a result of the NCVS. We present results from two experiments designed to test how the NCVS is produced and perceived. In the first experiment, we present data from an articulatory and acoustic analysis of the production of fronted /ɑ/ and /ɔ/. We find that some speakers distinguish /ɔ/ from /ɑ/ with a combination of both tongue position and lip rounding, while others do so using either tongue position or lip rounding alone. For speakers who distinguish /ɔ/ from /ɑ/ along only one articulatory dimension, /ɑ/ and /ɔ/ are acoustically more similar than for speakers who produce multiple articulatory distinctions. While all three groups of speakers maintain some degree of acoustic contrast between the vowels, the question is raised as to whether these articulatory strategies differ in their perceptibility. In the perception experiment, we test the hypothesis that visual speech cues play a role in maintaining contrast between the two sounds. The results of this experiment suggest that articulatory configurations in which /ɔ/ is produced with unround lips are perceptually weaker than those in which /ɔ/ is produced with rounding, even though these configurations result in acoustically similar output. We argue that these findings have implications for theories of sound change and variation in at least two respects: (1) visual cues can shape phonological systems through misperception-based sound change, and (2) phonological systems may be optimized not only for auditory but also for visual perceptibility.

## 1. Introduction

What are the driving forces of sound change? Most research on sound change and variation has focused on the acoustic and auditory properties of speech. For instance, misperception of the acoustic signal plays a central role in listener-based theories of sound change (Ohala, 1993; Blevins, 2004), while teleological models of phonology propose that speakers optimize their speech for auditory perceptibility and articulatory ease (Lindblom, 1990; Lindblom et al., 1995; Hayes et al., 2004). Although listeners have been shown to be sensitive to a wide range of non-auditory perceptual modalities (Sumby and Pollack, 1954; McGurk and MacDonald, 1976; Fowler and Dekle, 1991; Gick and Derrick, 2009; Mayer et al., 2013), the role of such cues in facilitating or inhibiting sound change has not been considered until recently (Johnson et al., 2007; McGuire and Babel, 2012; Johnson, 2015).

This paper investigates the role of visual cues in sound change by focusing on the audiovisual perception of interspeaker articulatory variation in the Northern Cities Vowel Shift (NCVS), a pattern of sound change observed in the dialect of English spoken in the Great Lakes region of the United States. Interspeaker articulatory variation, in which speakers employ differing articulatory strategies to achieve the same acoustic output, has been widely documented for sounds including /ɹ/ (Delattre and Freeman, 1968) and /s/ (Bladon and Nolan, 1977) in English. /ɹ/, for example, can be produced with a variety of tongue shapes broadly classified as “bunched” or “retroflex,” while /s/ can be produced with either an apical or a laminal articulation. This sort of variation has recently been argued to be a contributing factor to sound change, either because of its effects on patterns of coarticulation (Baker et al., 2011) or because of audiovisual perceptual properties that make visibly variable articulations perceptually less robust (McGuire and Babel, 2012). However, the forces governing articulatory variation are not entirely understood—what factors determine which articulatory strategy a speaker uses when the same acoustic output can be the result of multiple articulatory configurations? We suggest that articulatory variation may be restricted in part by the integration of visual cues in speech perception; although two articulatory configurations may have acoustically similar output, they may differ in their visual perceptibility. In this situation, language learners might prefer configurations that offer both auditory and visual contrast, as opposed to auditory contrast alone. On the other hand, if learners acquire an articulatory variant that is visually less distinct, sound change may occur as a result of misperception. This paper reports the results of two experiments investigating the hypothesis that visual speech cues restrict the ways in which articulatory patterns vary between speakers, thereby guiding the course of sound change and shaping phonological inventories.

The paper is organized as follows. In section 2, we review factors in articulation and perception that are argued to contribute to sound change and introduce relevant patterns of articulatory variation and multimodal speech perception. Section 3 provides an overview of the NCVS. An articulatory study of the vowels /ɑ/ and /ɔ/, as produced by speakers from Metro Detroit, is presented in section 4. Participants in this study are observed to vary with respect to how these vowels are produced in terms of both articulation and acoustics. In section 5, we present the results of an experiment investigating the role of visual speech perception in maintaining the contrast between these two sounds. Section 6 provides discussion of the results and considers the implications of these findings for theories of speech production and sound change.

## 2. Background

Perceptual factors have long been argued to play a central role in sound change. One of the predominant theories of sound change is that of Ohala (1981, 1983, 1989, 1993), who argues for a listener-oriented theory of sound change. Under this approach, the primary mechanism of sound change is the “innocent misapprehension” of ambiguous acoustic signals on the part of the listener, who subsequently maps the ambiguous signal onto a new phonological category. In the classic example, Ohala considers the effects of coronal consonants on a neighboring back vowel, e.g., /ut/. Because the articulation of a coronal consonant requires movement of the tongue toward the front of the oral cavity, neighboring vowels exhibit unusually high values of F2, such that /ut/ is realized as [yt]. Under normal circumstances, listeners expect this sort of coarticulatory effect and correct for it, mapping the acoustically ambiguous [yt] signal onto an /ut/ percept. However, listeners who fail to perform such correction, either because the coarticulatory source is weakened in the online channel or because the listener lacks experience with the language (as in the process of language acquisition), will map the [yt] signal onto its own phonological category, /yt/. The listener-turned-speaker subsequently produces /yt/ as the target articulation, which can be described as sound change within a single speaker. While the question of how such a change proceeds across a speech community remains open (the “actuation problem” of Weinreich et al., 1968), this view of misperception-driven sound change has proven to be a popular framework for laboratory investigations of language change and has been used to explain a number of cross-linguistically common patterns of sound change. For instance, Krakow et al. (1988) argue that listeners confronted with allophonically nasalized vowels are liable to misperceive the height of the vowel, but only when the conditioning nasal is weakened or deleted. Guion (1998) argues that the cross-linguistic frequency of velar palatalization is the result of perceptual reanalysis of velars as palatoalveolars, which are acoustically similar when appearing before a front vowel.

Arguing along similar lines, Blevins (2004, 2006) proposes a theory of Evolutionary Phonology in which sound change is viewed as the result of imperfect transmission across a noisy channel. On this account, sound change is primarily caused by listener-based reinterpretation, especially when the perception of a target sound is perceptually confusable, or when a listener chooses an underlying representation which differs from that of the speaker. Blevins argues that the tendency for languages to exhibit phonetically natural sound systems is best viewed in terms of Darwinian evolution: sounds (or sequences of sounds) that are easy to perceive or to produce are less likely to be misinterpreted by the listener, while difficult sounds are more likely to be misinterpreted and therefore more likely to undergo change. Like random mutations that occur in the replication of DNA, individual sound changes are not necessarily optimal in terms of phonetic naturalness, but only those changes that favor ease of perception or production are likely to be transmitted to subsequent generations of speakers. Moreover, speakers do not optimize their phonological grammars for phonetic naturalness; markedness effects are instead explained as the consequences of imperfect transmission of the speech signal. Similar argumentation is put forth by proponents of Substance-free phonology, who argue that markedness effects observed in synchronic phonological grammars are “epiphenomenal,” emerging as the result of phonetically natural diachronic sound changes rather than from optimization on the part of the language user (Hale and Reiss, 2008).

While Ohala and Blevins take the listener to be the central factor in determining the outcomes of phonetic change, functional or teleological models of phonology (Grammont, 1939; Vennemann, 1988; Lindblom, 1990) stress the importance of speakers attempting to make themselves understood even under adverse communicative conditions. One such model is that of Lindblom (1990), who proposes a theory of hyper- and hypo-articulation (H&H Theory) to explain online phonetic variation in speech production. Under this model, speakers alter their production targets on the basis of both internal, system-oriented goals, as well as external, output-oriented goals. These goals are in perpetual competition, trading off in response to the speaker's articulatory desires and to the speaker's estimation of the listener's perceptual requirements. In terms of speech production (and in motor control more generally), Lindblom observes that speakers tend to hypoarticulate, exerting the minimal amount of articulatory effort necessary to achieve sufficient contrast in a given environment. However, when required by perceptual demands, speakers hyperarticulate in order to optimize their speech for maximum perceptibility. Under this sort of approach, marked or perceptually weak sound patterns are predicted to be more likely to undergo change than perceptually robust or unmarked patterns, as a result of reanalysis on the part of the language learner.

Taken together, the listener-oriented and speaker-oriented approaches sketch a view in which the listener may misperceive the phonetic signal transmitted by the speaker, resulting in sound change, but where language users also incorporate knowledge of variability into their phonetic representations. The listener-turned-speaker can exploit this variability in order to optimize their pronunciations for perceptibility as well as for articulatory effort. This is the view put forth by Lindblom et al. (1995), who acknowledge that sound changes *can* occur as a result of misperception, but argue for a stronger role of the speaker in evaluating and selecting phonetic variants for production. They note that correct perception of a linguistic message relies not only on the phonetic signal actually produced by the speaker, but also on the listener's expectations of that message. For instance, factors including syntactic knowledge, lexical frequency, and sociophonetic knowledge (see, e.g., Sumner and Samuel, 2009) make certain words more predictable than others. Thus, when a predictable word is pronounced in a novel way, the listener correctly perceives the speaker's message, yet is aware that the pronunciation of that word differed from the standard pronunciation. The listener-turned-speaker evaluates this novel variant for its articulatory and perceptual fitness, and may choose to adopt it as a new pronunciation norm. Thus, they argue that sound change is not purely the result of accidental misperception, but that sound changes can also be adaptive.

Nevertheless, a crucial component of both approaches to sound change is that the mechanisms of conversational interaction give rise to a wide range of phonetic variation (Ohala 1989's “pool of synchronic variation”) that provides the foundation upon which diachronic sound changes are built. Until recently, however, most work on variation and change has centered around phonetic precursors to sound change in the acoustic and auditory domains. Variation in the articulatory domain is typically considered only insofar as it results in acoustic ambiguities. Yet, a wealth of evidence suggests that sounds can vary not only in their acoustics, but also in their articulation; two sounds that have the same acoustic output can be produced with distinct articulatory configurations. Such variation frequently arises in response to coarticulatory demands (Perkell et al., 1993; Stone and Vatikiotis-Bateson, 1995), but can also be observed as categorical differences between speakers or as an allophonic pattern within the speech of a single speaker (Mielke et al., 2010). Because such articulatory variants have similar acoustic outputs, they are often considered to be imperceptible to listeners; however, recent research has shown that articulatory variation can be a trigger for sound change due to coarticulatory effects (Baker et al., 2011) or audiovisual perceptual properties (McGuire and Babel, 2012).

The most well known example of interspeaker articulatory variation is perhaps the American English post-alveolar approximant /ɹ/. This sound is traditionally described as having two variants, retroflex and bunched (Uldall, 1958), although more fine-grained classifications have been proposed (Delattre and Freeman, 1968; Espy-Wilson, 2004). Despite the wide range of articulatory configurations for /ɹ/, the acoustic realization is largely consistent across variants such that /ɹ/ exhibits an unusually low F3 that approaches F2 (Espy-Wilson, 1987; Hagiwara, 1995; Westbury et al., 1995; Stevens, 2000). Espy-Wilson (2004) and Zhou et al. (2008) demonstrate that variants of /ɹ/ are similar in F1–F3, but that subtle differences exist in F4 and F5. Evidence from perceptual studies, however, suggests that listeners cannot reliably distinguish between articulatory variants (Twist et al., 2007).

One study of articulatory variability of particular interest for the present study is presented by De Decker and Nycz (2012). De Decker and Nycz conducted an ultrasound study of [æ]-tensing in the Mid-Atlantic variety of American English, as spoken in New Jersey. They observe variation in how speakers produce the contrast between tense and lax [æ]. While some speakers produce the contrast with a difference in tongue position, other speakers produce tense and lax [æ] with identical tongue positions. Given that these speakers continue to produce an acoustic tensing distinction, De Decker and Nycz suggest that the contrast may be maintained by nasalization rather than by tongue position. Because movements of the velum and tongue dorsum are not visible to listeners, it is likely that both articulatory strategies are perceptually equivalent.

Interestingly, however, not all logically possible patterns of articulatory variation are attested. Harrington et al. (2011), for instance, have shown that speakers of Standard Southern British English achieve /u/-fronting entirely through tongue repositioning, rather than through a reconfiguration of the lips, even though both articulations can produce an increase in F2. Evidence for the roundedness of /u/ in Standard Southern British English comes from a variety of experimental data. In an acoustic study, Harrington et al. show that /u/ exerts a coarticulatory lowering effect on the spectrum of a preceding /s/. In a visual perception study, they show that German speakers were more likely to identify a token of /u/ in a silent video as /u/, /y/, or /o/ than as an unround vowel. Finally, in an EMMA analysis of /u/, they show that the tongue position for /u/ is closer to /i/ than to /ɔ/, while the degree of lip protrusion is closer to /ɔ/ than to /i/. Thus, Harrington and colleagues conclude that in the process of fronting, /u/ has retained its rounding. This was the case for all speakers in their study, despite the fact that variation is in principle possible given the potential for a trading relation between tongue position and lip configuration.

While there are likely many factors that contribute to the range of possible articulatory variation, including physiological differences between speakers (Brunner et al., 2009; Bakst and Lin, 2015) and differences in auditory/haptic acuity (Gluth and Hoole, 2015), one factor which might plausibly restrict the range of possible articulatory variants is a sound's audiovisual perceptual properties. Beginning in the mid twentieth century, a wealth of experimental evidence demonstrated that visual cues can enhance auditory speech perception and even override the acoustic signal under certain conditions. In one early demonstration of audiovisual integration in speech perception, Sumby and Pollack (1954) tested lexical identification under both auditory and audiovisual conditions, with stimuli presented with varying degrees of white noise. They found that participants excelled at identification in both conditions when levels of noise were low, but that performance diverged as the level of noise was increased. Rates of identification under noisy conditions were higher in the audiovisual condition, demonstrating that visual cues can enhance speech perception.

McGurk and MacDonald (1976) describe perhaps the most well known example of audiovisual speech perception, the McGurk Effect. McGurk and MacDonald find that when an auditory stimulus is paired with incongruous video from another stimulus, participants perceive the sound as a fusion between the auditory and visual channels, an effect that persists even among listeners who are aware of the incongruity. Notably, however, fusion does not occur when the auditory channel is paired with video of a labial segment; when auditory [ga] is paired with visual [ba], the resulting percept is [ba] or [bga]. This finding demonstrates that the presence of a visible labial gesture forces perception of a segment as labial, while less-visible lingual articulations are susceptible to fusion or misperception.

Similar results were obtained by Braida et al. (1998), who tested the audiovisual perception of stop place among speakers of Japanese. In an audio-only condition, participants exhibited a high rate of misidentification of stops at labial, coronal, and dorsal places of articulation, such that stimuli containing [b] were perceived as labial in only 56% of cases. In the visual condition, however, stimuli containing [b] were correctly identified as labial in 98% of trials, while stimuli containing [d] or [g] were identified as labial in <2% of trials. Moreover, a high degree of misperception between [d] and [g] was observed in both auditory and visual conditions.

With respect to vowel perception, Traunmüller and Öhrström (2007a,b) find that listeners rely heavily on visual cues in perception of the Swedish /i/-/y/ rounding contrast and argue that acoustic cues alone are insufficient to distinguish these vowels. When Swedish speakers were presented with incongruous audiovisual stimuli in which an unround auditory stimulus was paired with a round visual stimulus, participants perceived the vowel as round in as many as 99% of trials. When auditorily round vowels were presented with video of unround vowels, the vowel was perceived as round. These findings suggest that visual cues can be sufficient for maintaining an otherwise perceptually weak contrast.

More recently, a series of studies by Ménard et al. (2009, 2013, 2015, 2016) has demonstrated the importance of audiovisual perception to speech intelligibility through an investigation of differences in the use of visible articulation by sighted and congenitally blind speakers. Ménard et al. (2009), for instance, tested the production and discrimination of vowel contrasts among sighted and blind speakers of Canadian French. They find that blind speakers exhibit greater auditory discrimination abilities than sighted speakers, but that sighted speakers have a significantly larger vowel space, suggesting that the availability of visual speech cues (or lack thereof) influences speakers' production targets. Ménard et al. (2015, 2016) find that in producing clear speech, only sighted speakers produce more pronounced lip movements, while blind speakers rely on changes in tongue movement. Thus, when attempting to enhance speech intelligibility, sighted speakers seem to consider how their speech will be perceived not only auditorily, but also visually.

Beyond audiovisual perception, researchers have demonstrated that numerous other perceptual modalities influence human speech perception. Fowler and Dekle (1991) tested whether a listener's perception is influenced by haptic cues perceived by placing their hands on the speaker's face and neck. They find that [ba/ɡa] auditory stimuli are more likely to be perceived as [ba] when paired with a haptically-perceived lip closure. Gick and Derrick (2009) observe that speech perception is influenced by the integration of aerotactile cues, such that applying a puff of air to a listener's skin increases the likelihood of perceiving unaspirated /b/ as aspirated /p/. Mayer et al. (2013) demonstrate that listeners are sensitive even to relatively indirect speech cues: listeners are more likely to perceive a sound as aspirated when it is paired with video of a flickering candle than when it is paired with video of a candle with a still flame.

Despite the well known influence of visual and other non-auditory cues on speech perception, however, few studies have considered whether visual speech perception may play a role in misperception-based sound change. Johnson et al. (2007) investigated excrescent nasals as found in the Toulouse variety of French, where standard [sav$\tilde{ɔ}$] is realized as [savɔŋ]. They suggest that nasalized vowels alternate with velar nasals, rather than labial or coronal nasals, due to the visual similarity between velar consonants and vowels. Johnson (2015) investigates the hypothesis that stop debuccalization can occur as the result of temporal misalignment of auditory and visual speech cues. While he finds only a small effect of visual influence on perception, he suggests that audiovisual integration may provide a push toward debuccalization of labial stops, but that the process ultimately depends on other phonetic processes. Most relevant to the present study, McGuire and Babel (2012) argue that visual cues are responsible for asymmetry in changes involving /θ/ and /f/. Whereas /θ/ > /f/ is a common sound change, /f/ > /θ/ changes are typologically rare, a tendency they attribute to a wider range of articulatory variability observed for /θ/ than for /f/. While /f/ is uniformly produced with a labiodental articulation, American English /θ/ exhibits variation such that it can be produced with a dental or interdental articulation. Such variability makes /θ/ less visibly distinct than /f/ and therefore more likely to be misperceived.

Although it has been broadly demonstrated that listeners are sensitive to a wide variety of non-auditory perceptual cues, only a small number of studies have considered whether such cues contribute to the development of phonological systems. In the case of articulatory variation in particular, it may be the case that visual speech perception restricts the range of possible variants, either by providing language learners with a strong cue to how a given sound is articulated, or by making contrasts between visually distinct sounds easier to perceive. In this paper, we test the hypothesis that visual speech cues influence the direction of sound change by reducing the likelihood of misperception of the speech signal. Specifically, we hypothesize that visual speech cues may help listeners to identify acoustically similar sounds, thereby avoiding merger.

## 3. The northern cities vowel shift

**Figure 1:**
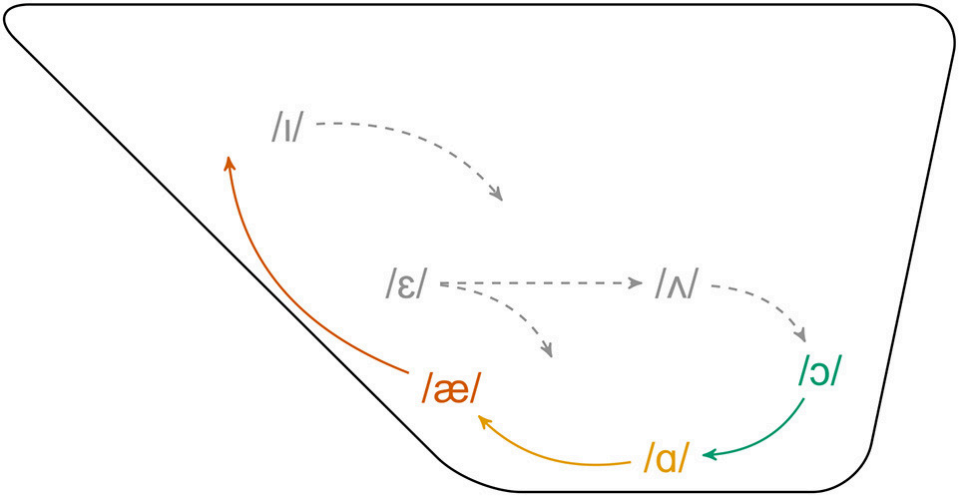
Schematic diagram of the Northern Cities Vowel Shift. Solid, colored lines indicate early stages, dashed gray lines indicate later stages (Adapted from Labov et al., 2006, p.190).

The NCVS, observed in the Great Lakes region of the United States (the Inland North), is one of the most widely studied sound changes in the sociolinguistic literature. As a chain shift, the NCVS involves the coordinated movement of several vowels, as observed in Figure 1. In the earliest stage of this shift, /æ/ is raised such that it can exhibit an F1 as low or lower than that of /ɪ/ (Labov, 1994). The raising of /æ/ creates an opening in the vowel space, which /ɑ/ moves forward to fill. Labov et al. (2006) find that /ɑ/ exhibits a mean F2 of >1,450 Hz among Inland North speakers. In contrast Peterson and Barney (1952) found a mean F2 for /ɑ/ of 1,220 Hz for women and 1,090 Hz for men in a study of the General American dialect. Following the fronting of /ɑ/, /ɔ/ moves forward to adopt the former position of /ɑ/. Later stages of the chain shift involve the movement of several additional vowels: /ʌ/, /ɛ/, and /ɪ/.

Like most sociolinguistic phenomena, descriptions of the NCVS are based almost entirely on acoustic measurements.^1^ The fronting of /ɑ/ and /ɔ/, for instance, is described as an increase in the value of F2. As in the case of /u/-fronting described above, however, an increase in F2 can be the result of any gesture that shortens the vocal tract, including both tongue fronting and lip unrounding. As such, it can be problematic to make inferences about the articulation of vowel fronting on the basis of acoustic measurements alone. Assuming that speakers with the NCVS do not merge /ɑ/ and /ɔ/, there exist three possible articulatory strategies when it comes to the fronting of /ɔ/. First, the tongue position for /ɔ/ may move forward, approaching that of /ɑ/, while the lips remain round. A second possibility is that /ɔ/ becomes unround with no change in tongue position. Third, these strategies may be combined such that speakers produce fronted /ɔ/ with some degree of lip unrounding and some fronting of the tongue.

Majors and Gordon (2008) used video recording to perform an analysis of lip unrounding in two speakers from St. Louis, where the NCVS is in effect to some extent. Majors and Gordon find that /ɔ/ can be fronted while retaining its rounding, suggesting that /ɔ/-fronting and lowering in the NCVS may be accomplished through a repositioning of the tongue alone. This result is similar to the findings of Harrington et al. (2011), described above, who found that /u/-fronting in Standard Southern British English is achieved by tongue fronting, rather than by lip unrounding. However, because video analysis only allows for measurement of labial articulation, Majors and Gordon are unable to consider the actual behavior of the tongue in producing these sounds. In addition, St. Louis is the least consistent of the Inland North cities in terms of the number of NCVS-related changes and the number of speakers exhibiting the shift (Labov et al., 2006), so the patterns observed in St. Louis may differ from those found in more typical cities such as Chicago or Detroit. As such, there is strong motivation for considering the articulation of the NCVS among speakers from one of these cities. In order to address this question, an articulatory-acoustic study of the NCVS was conducted for speakers from Metro Detroit. Results from this experiment are described in the following section.

## 4. Experiment 1: production of the NCVS

### 4.1. Methods and materials

#### 4.1.1. Participants

Eight speakers participated in the production experiment. Participants included five men (ages 24–29) and three women (ages 22, 23, and 39), all of whom were born and raised in Metro Detroit until at least the age of 18.^2^ Seven of the eight speakers resided in the Washington, DC region at the time of the experiment, while Speaker 1 resided in Metro Detroit.^3^ Two of the eight speakers were excluded from analysis. The first, a 29 year-old man, was excluded because of poor ultrasound imaging that prevented accurate tracking of tongue contours. The second, a 25 year-old man, was excluded because his vowel production was not consistent with the NCVS in that /ɑ/ did not exhibit the characteristically high F2 associated with the shift. Articulatory data from this speaker would therefore reveal little with respect to the behavior of fronted /ɑ/ and /ɔ/. In total, data from six speakers are considered in the analysis.

#### 4.1.2. Procedure

Participants were asked to repeat a wordlist containing 100 monosyllabic words of English, including 20 words for each of the vowels /i/, /u/, /æ/, /ɑ/, and /ɔ/. The target vowels were the low back vowels /ɑ/ and /ɔ/, while /i/ and /u/ were included to serve as reference points for lip spread and lip openness, respectively. A subset of /u/ words containing the sequence /ul/ were included to serve as a reference point for tongue backness. Finally, /æ/, which is the vowel argued to have initially triggered the chain shift, was included because it forms the basis for one of the metrics established by Labov et al. (2006) to measure degree of participation in the NCVS. Words were embedded in the carrier phrase “say _____ again” and presented to participants in pseudo-random order.

Recording took place in a sound-attenuated booth at Georgetown University. Ultrasound data were captured using a SonoSite M-Turbo portable ultrasound machine with a C60x 5–2 MHz transducer set to a scan depth of 9.2 cm. Ultrasound images were synchronized with the audio stream using an Elgato Video Capture device, which recorded the NTSC output of the ultrasound machine at a resolution of 640 × 480 pixels at 30 frames per second (fps). To allow for the comparison of tongue contours across tokens, the ultrasound transducer was kept stable by attaching it to an articulated arm (Manfrotto Magic Arm) that was mounted to a table in front of the participant. Head movement was mitigated with a chair-mounted headrest (cf. Stone et al., 1988). Audio was recorded with a Shure SM58 cardioid microphone and an Olympus LS-100 solid state recorder. Video of the speaker's lips was captured using a Canon XA10 camcorder at a resolution of 1,920×1,080 pixels at 30 fps. The camera was positioned approximately 1.5 m in front of the speaker.

#### 4.1.3. Data analysis

LPC formant measurements were taken in Praat (Boersma and Weenink, 2016). Except for tokens containing /æ/, measurements were taken at the point of F1 maximum. For /æ/, the point of F2 maximum was used, as suggested by Labov et al. (2006, 38). Vowel formant measurements were normalized using the log-mean normalization formula used for the *Atlas of North American English* (*ANAE*; Labov et al., 2006, 39–40), as implemented in the R package vowels (Kendall and Thomas, 2014), and converted to Bark scale. While this type of normalization is typically best suited for larger sample sizes, the *ANAE* method was chosen in order to allow the formant values obtained in this study to be reasonably compared to the values found for the Inland North speakers in *ANAE*. As such, the metrics established by Labov et al. (2006) to measure a speaker's degree of participation in the NCVS can be applied to the speakers in this study.

Still frames corresponding to the formant measurement points were extracted from the ultrasound and video recordings using the CV2 module in Python and saved as JPEG images.^4^ Extracted video frames were analyzed using the vector graphics editor Inkscape. A box was drawn around the speaker's lips such that the horizontal lines were tangential to the upper and lower vermillion borders, and the vertical lines were tangential to the left and right commissures. A Python script was used to extract the horizontal and vertical dimensions of the box in pixels from the vector graphics file. Lip measurements were converted to centimeters based on a ruler held against the speaker's lips at the start of recording.

**Figure 2:**
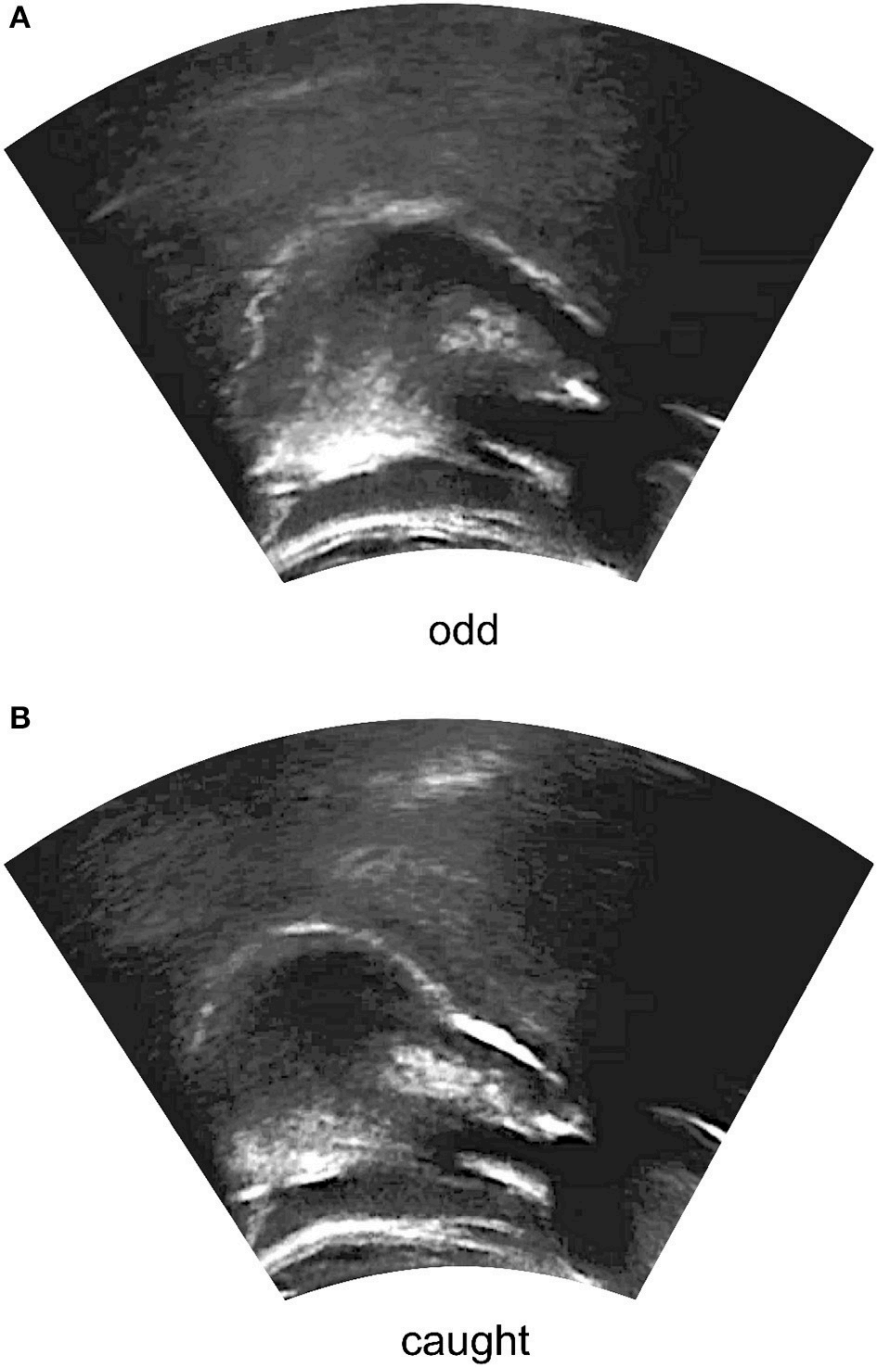
Extracted midsagittal ultrasound frames for the tokens **(A)** odd and **(B)** caught, as produced by Speaker 6. The right side of the image corresponds to the front of the mouth. The tongue surface is visible as the white line near the center of the image. The dark portions on the left and right sides of the image are the shadows of the hyoid and the mandible.

Ultrasound frames, like those shown in Figure 2, were imported into EdgeTrak (Li et al., 2005), which was used to generate contour data for each token. Several points were placed manually along the lower edge of the visible tongue surface, and the Optimize function was used to improve the fit of the contour to this edge. From this contour, EdgeTrak was set to extrapolate a total of 100 points along the tongue surface, which were exported and analyzed using smoothing spline analysis of variance (SSANOVA; Gu 2002). SSANOVA is a statistical method for determining whether significant differences exist between best-fit smoothing splines for two or more sets of data. It has been used in linguistic research to analyze both ultrasound tongue contour data (Davidson, 2006; Chen and Lin, 2011; De Decker and Nycz, 2012; Lee-Kim et al., 2013, 2014) and formant measurements over time (Baker, 2006; Nycz and De Decker, 2006; Fruehwald, Unpublished Manuscript). Here, the SSANOVA model was generated using the ssanova function of the gss package for R (Gu, 2014; R Core Team, 2016).^5^

### 4.2. Results

Based on canonical descriptions, /ɑ/ and /ɔ/ are expected to differ along two articulatory dimensions. /ɑ/ should be articulated with a low, pharyngealized tongue position and unround lips, while /ɔ/ should have a somewhat higher tongue position and the addition of lip rounding. For Inland North speakers, however, both vowels exhibit an F2 that is higher than in most other dialects of North American English. For such a change to occur, the tongue position for /ɑ/ must move forward, given that it is already unround (setting aside the possibility of an increase in lip spread). As noted above, however, several articulatory strategies exist that might increase the F2 of /ɔ/: the tongue can front, such that /ɑ/ and /ɔ/ are contrasted by lip rounding, the lips can unround, such that /ɑ/ and /ɔ/ are contrasted by tongue position, or speakers might produce a contrast between /ɑ/ and /ɔ/ through both tongue position and lip rounding. In this experiment, all three configurations are observed. The following sections present representative articulatory data from a speaker exhibiting each of these patterns before turning to an analysis of the effect of these articulatory differences on the acoustic signal.

#### 4.2.1. Articulatory results

**Figure 3:**
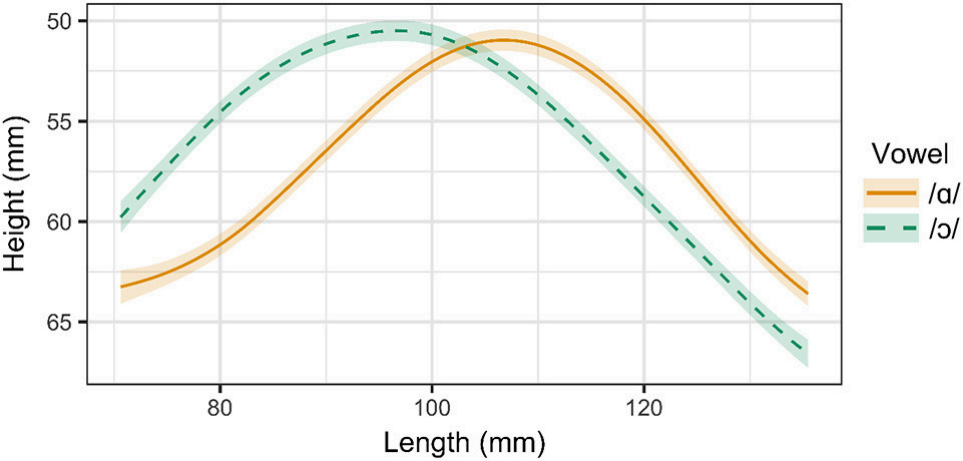
Main effect curves for SSANOVA model of /ɑ/ and /ɔ/ for Speaker 1. Shading indicates 95% Bayesian confidence interval.

Three of the six speakers examined in this experiment distinguish between /ɑ/ and /ɔ/ through both tongue position and lip rounding. These speakers include Speaker 1 (male, 26), Speaker 2 (male, 26), and Speaker 3, (female, 39). Tongue contours for Speaker 1 are presented in Figure 3. As in the ultrasound images in Figure 2, the right side of the contour corresponds to the tongue front, while the left side corresponds to the tongue root. The shaded regions surrounding the smoothing spline estimates for each vowel represent the 95% Bayesian confidence intervals returned by the SSANOVA model. Overlap between the confidence intervals for the two contours indicates that the difference between the contours is not significant in that particular region of the tongue. For these speakers, the tongue contours for /ɑ/ and /ɔ/ differ significantly from one another along the entire length of the tongue, providing evidence that these vowels are produced with distinct tongue positions. The constriction for /ɑ/ is higher and more front than that of /ɔ/, which exhibits a greater degree of pharyngeal constriction.

**Figure 4:**
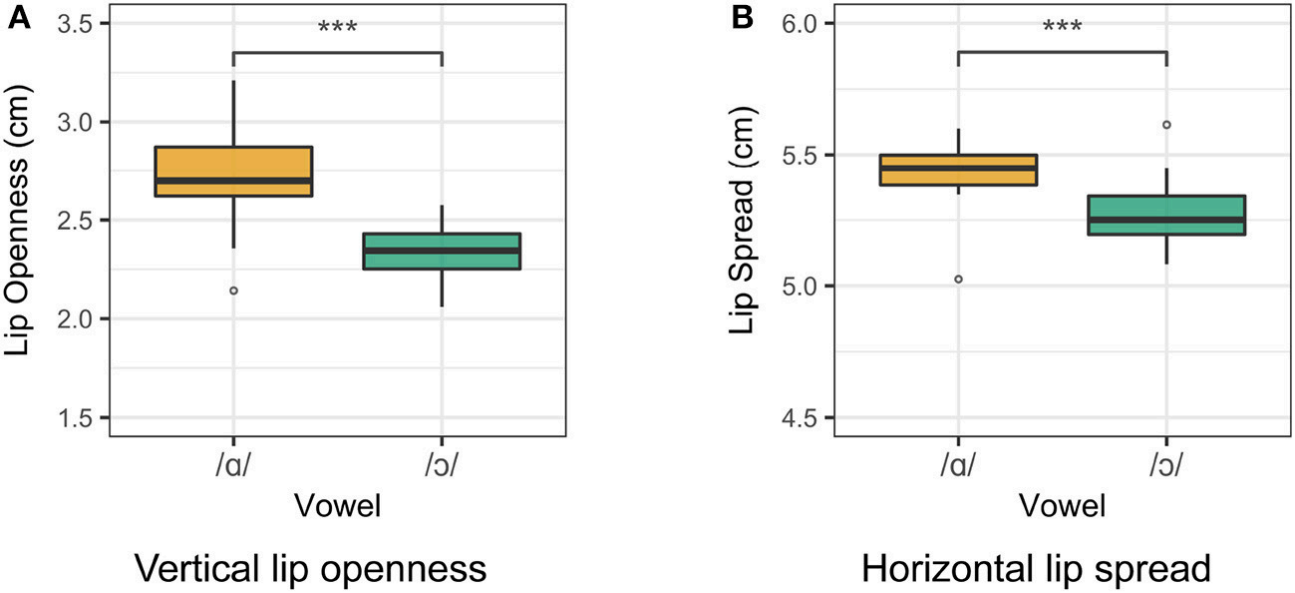
Lip measurements for Speaker 1. Smaller measurements indicate a greater degree of lip rounding. **(A)** Vertical lip openness. **(B)** Horizontal lip spread.

Lip rounding measurements for Speaker 1 are presented in Figure 4. For both vertical lip openness and horizontal lip spread, a smaller value indicates a greater degree of lip rounding. The degree of lip openness and lip spread was measured for each speaker. Two one-way ANOVA tests were run for the vertical and horizontal lip measurements for all vowels for each speaker. For Speaker 1, vowel class is a significant predictor of both lip openness [*F*_(4, 95)_ = 74.5, *p* < 0.001] and lip spread [*F*_(4, 95)_ = 63.4, *p* < 0.001]. The difference between /ɑ/ and /ɔ/ in both lip spread and lip openness is significant (*p* < 0.001), as revealed by a Tukey *post-hoc* test. For Speakers 2 and 3, /ɑ/ and /ɔ/ differ significantly in lip openness, but not in lip spread. Although some languages do contrast distinct types of labialization (Lindau, 1978; Linker, 1982), it is assumed for the purposes of this study that both dimensions of labial opening are correlates of the feature [round], and a significant difference in either measure is treated as a rounding contrast.

**Figure 5:**
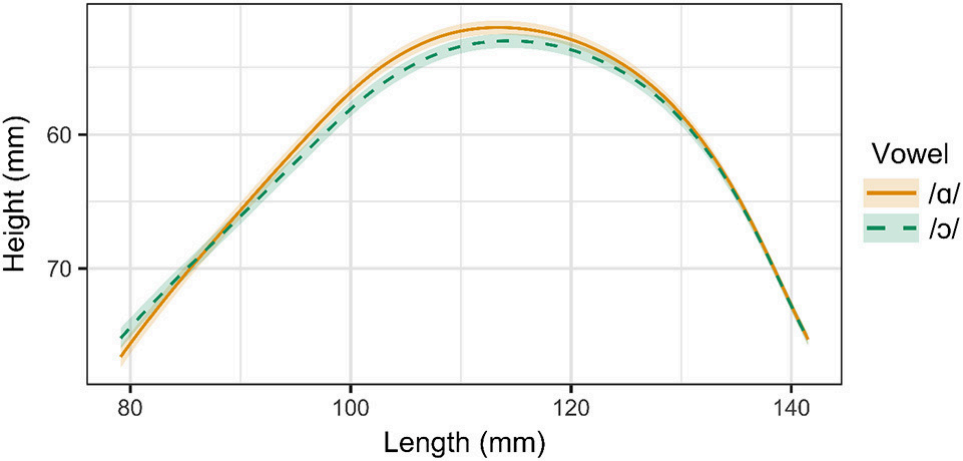
Main effect curves for SSANOVA model of /ɑ/ and /ɔ/ for Speaker 4. Shading indicates 95% Bayesian confidence interval.

**Figure 6:**
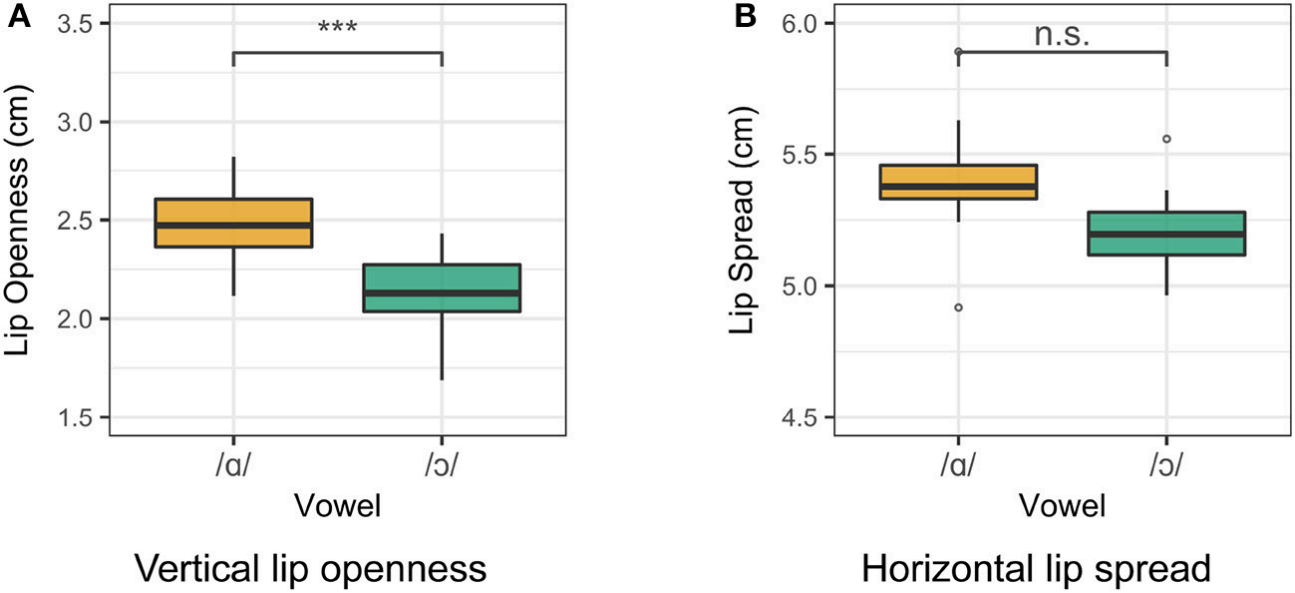
Lip measurements for Speaker 4. Smaller measurements indicate a greater degree of lip rounding. **(A)** Vertical lip openness. **(B)** Horizontal lip spread.

For Speakers 4 and 5, /ɑ/ and /ɔ/ differ in lip rounding but not in tongue position. Smoothing spline estimates for /ɑ/ and /ɔ/ as produced by Speaker 4 are presented in Figure 5. Except for a small region near the tongue dorsum, the smoothing splines for /ɑ/ and /ɔ/ do not differ significantly. In the dorsal region, the tongue position for /ɔ/ is actually anterior to that of /ɑ/, which is expected to result in a higher F2 for /ɔ/ than for /ɑ/, which is not the case. However, this speaker maintains the contrast between /ɑ/ and /ɔ/ through lip openness, as observed in Figure 6. Vowel class is a significant predictor of lip openness [*F*_(4, 95)_ = 35.93, *p* < 0.001] and lip spread [*F*_(4, 95)_ = 27.8, *p* < 0.001]. Tukey *post-hoc* test results show that /ɑ/ and /ɔ/ differ significantly in lip openness, but not in lip spread. The opposite holds for Speaker 5, for whom /ɑ/ and /ɔ/ differ significantly in lip spread, but not in lip openness.

**Figure 7:**
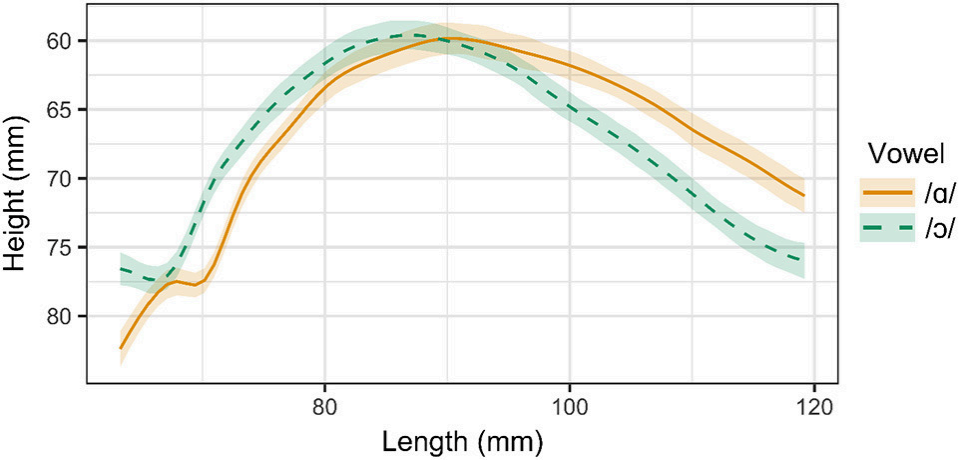
Main effect curves for SSANOVA model of /ɑ/ and /ɔ/ for Speaker 6. Shading indicates 95% Bayesian confidence interval.

**Figure 8:**
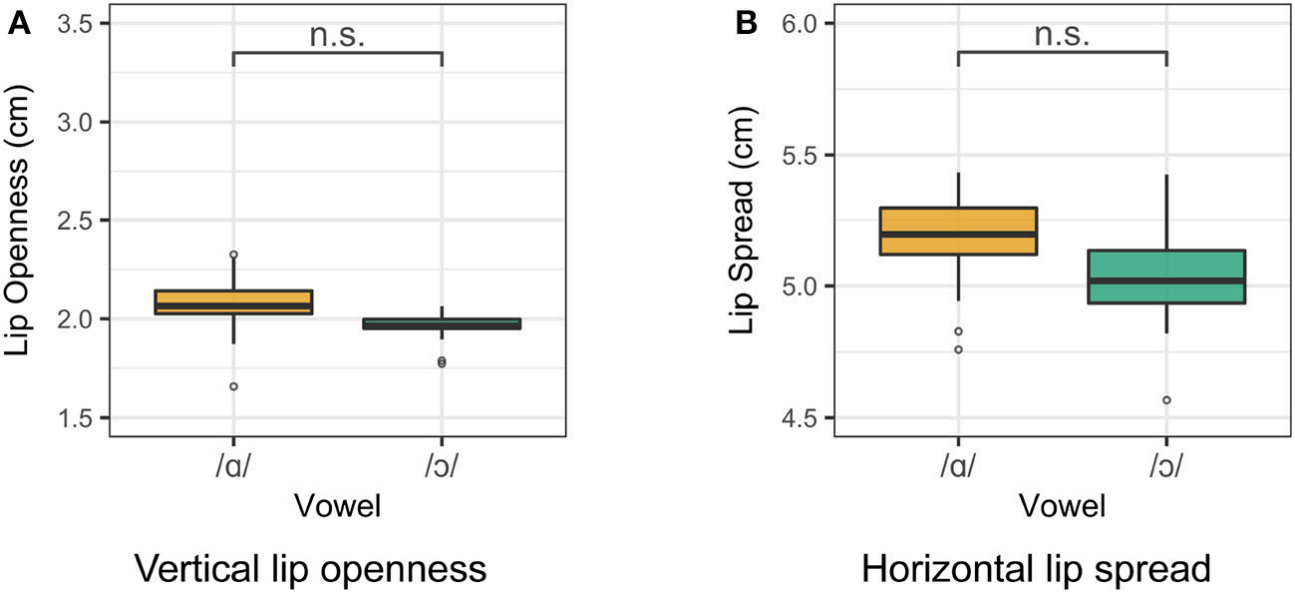
Lip measurements for Speaker 6. Smaller measurements indicate a greater degree of lip rounding. **(A)** Vertical lip openness. **(B)** Horizontal lip spread.

For Speaker 6, a 21 year-old woman, /ɑ/ and /ɔ/ differ in tongue position but not in lip rounding. Smoothing splines for /ɑ/ and /ɔ/ as produced by Speaker 6 are presented in Figure 7. For this speaker, tongue contours for /ɑ/ and /ɔ/ differ significantly throughout the tongue root and body. Lip measurement results for Speaker 6 are presented in Figure 8. For Speaker 6, vowel class is a significant predictor of both lip openness [*F*_(4, 93)_ = 13.74, *p* < 0.001] and lip spread [*F*_(4, 93)_ = 35.24, *p* < 0.001], but a Tukey *post-hoc* test reveals that /ɑ/ and /ɔ/ do not differ significantly in either measure.

The results of the articulatory analysis thus suggest that three distinct patterns exist among Inland North speakers, as shown in Table 1. In Pattern A, speakers exhibit a significant difference between /ɑ/ and /ɔ/ in both lip spread and tongue position, with Speaker 1 producing an additional distinction in lip openness. In Pattern B, neither Speaker 4 nor Speaker 5 produce a significant difference between /ɑ/ and /ɔ/ in tongue position. However, both speakers produce a significant contrast between these vowels in lip configuration, with Speaker 4 producing a lip openness contrast and Speaker 5 producing a lip spread contrast. While Speaker 4 and Speaker 5 differ with respect to the particular labial gesture used to distinguish /ɔ/ from /ɑ/, both speakers do in fact make a labial distinction between these vowels. Finally, in Pattern C, Speaker 6 produces a significant contrast in tongue position alone; for this speaker, the differences between /ɑ/ and /ɔ/ in both lip openness and lip spread fail to achieve significance.

**Table 1:** Summary of articulatory patterns observed in Experiment 1.

		**Gender**	**Age**	**Articulatory distinction**
Pattern A	Speaker 1	Male	26	Tongue and lip contrast
	Speaker 2	Male	29	
	Speaker 3	Female	39	
Pattern B	Speaker 4	Female	23	Lip contrast only
	Speaker 5	Male	26	
Pattern C	Speaker 6	Female	22	Tongue contrast only

#### 4.2.2. Acoustic results

**Figure 9:**
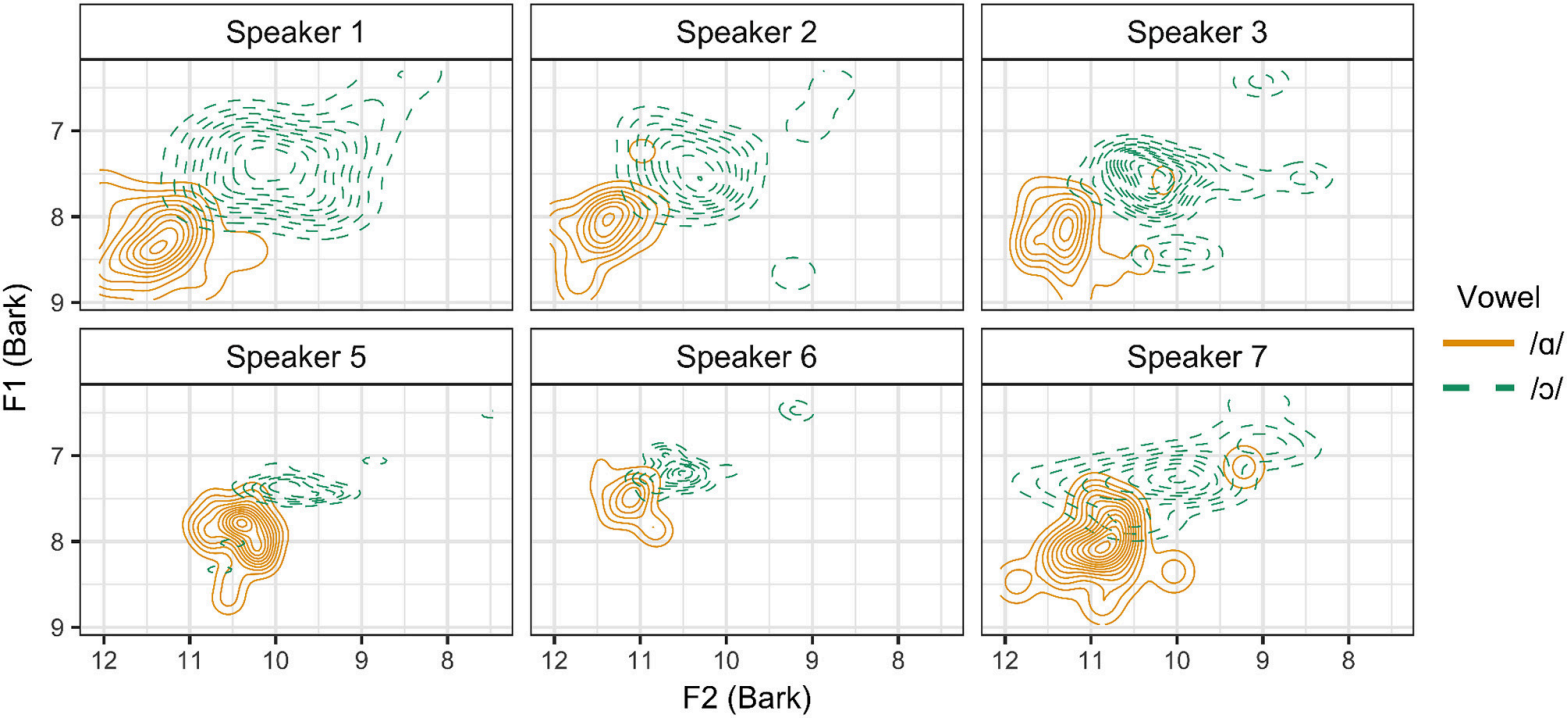
Kernel density estimation plot of normalized, Bark-scaled vowel formant measurements for /ɑ/ and /ɔ/ for all speakers.

In order to determine whether these three articulatory strategies differ in their acoustic output, vowel formant measurements were analyzed for each speaker. Normalized formant measurements are presented in Figure 9 as kernel density estimation plots of the distribution of /ɑ/ and /ɔ/ for each speaker in the F1 × F2 space.^6^ It is impressionistically observed that Speaker 1 has the widest distribution of tokens for /ɑ/ and /ɔ/, which may be accounted for by the fact that this speaker distinguishes between these vowels through tongue position, lip rounding, and lip spread. Speakers 5 and 6, both of whom produce no contrast between /ɑ/ and /ɔ/ in tongue position, appear to have the greatest amount of overlap in the distribution of these vowels.

The degree of overlap between these vowels was quantified by calculating a Pillai-Bartlett trace (“Pillai score”) for each speaker. A Pillai score is the output of a multivariate analysis of variance (MANOVA) model, which allow for statistical analysis of multiple dependent variables. This method returns a score ranging from 0 to 1, where 0 indicates that the two distributions are identical and where 1 indicates no overlap at all. It was first used in sociophonetic research by Hay et al. (2006), and has since been applied in the literature by Hall-Lew (2010) and compared to other methods of measuring vowel distance by Nycz and Hall-Lew (2014). In this case, the Pillai score was used to measure the difference between /ɑ/ and /ɔ/ in F1, F2, and F3, while taking into account the preceding and following consonantal environments. Unlike other measures of vowel distance, such as Euclidean distance, the Pillai-Bartlett trace takes into account not only the distance between the means of the vowel categories, but also the degree of overlap between distributions.

**Figure 10:**
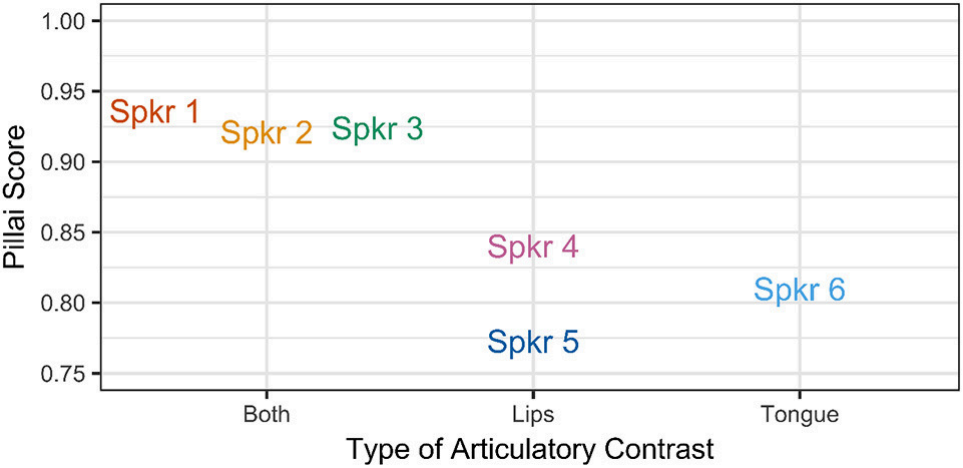
Pillai score for each speaker, by articulatory configuration.

The results are presented in Figure 10, where the Pillai score for each speaker is plotted with speakers grouped by articulatory pattern. Note that for all speakers, the Pillai score is >0.75, which is close to the maximum score of 1.00, indicating that these vowels are relatively distinct for all speakers. While Pillai scores are not necessarily comparable across studies, Nycz and Hall-Lew (2014) find Pillai scores of < 0.25 for speakers of Canadian and Scottish English, for whom /ɑ/ and /ɔ/ are merged. Hay et al. (2006) find that speakers of New Zealand English differ greatly in the degree of overlap for the vowels /iə/ (near) and /eə/ (square): the speaker with the least distinct vowels in their sample received a Pillai score of 0.0009, while the speaker with highest degree of contrast received a score of 0.969. Nevertheless, a clear pattern is observed for the speakers in the present study: the Pillai score is lower for speakers of Patterns B and C, who make use of only one articulatory gesture to distinguish between /ɑ/ and /ɔ/, than for speakers of Pattern A, who produce a contrast along multiple articulatory dimensions. This finding suggests that the use of only a single gesture to produce the /ɑ/-/ɔ/ contrast results in a greater degree of acoustic overlap.

### 4.3. Discussion

Despite a three-way pattern in articulatory strategy, a two-way pattern in the acoustic signal is observed. For speakers exhibiting both tongue position and lip rounding contrasts, the mean Pillai score is 0.927, indicating that these vowels are distinct. However, for speakers producing only one type of articulatory contrast, the mean Pillai scores are 0.806 for speakers producing a lip rounding contrast, and 0.809 for Speaker 6, who produces only a tongue position contrast. Although the difference is relatively small, these lower scores suggest that the distributions of /ɑ/ and /ɔ/ are more similar for speakers who produce these vowels using fewer articulatory gestures. It therefore appears that the use of additional articulatory gestures serves to enhance the acoustic contrast.

In this respect, the type of articulatory variation observed here differs from other instances of articulatory variability described in section 1, such as that for /ɹ/. Although small acoustic differences can be observed in the higher formants for variants of /ɹ/ (Delattre and Freeman, 1968; Espy-Wilson, 2004), this variability is generally suggested to be difficult to perceive (Twist et al., 2007). If the articulatory patterns observed in this experiment differ from one another in their perceptibility, it is possible that some articulatory patterns may lead to eventual merger of the two vowels, or that perceptually weak articulatory variants will be dispreferred. Because Patterns B and C both result in a smaller degree of acoustic contrast, it is reasonable to predict that both strategies should be perceptually weaker, if only marginally, than Pattern A. What is less clear, however, is whether Patterns B and C differ perceptually from one another. Although both patterns result in a similar degree of acoustic contrast between the two vowels, they differ crucially in that Pattern B retains lip rounding, which is visible, while speakers exhibiting Pattern C maintain the contrast through tongue position, which is less visible. As discussed in section 1, visual speech cues are known to influence patterns of speech perception; it is possible that such cues play a role in maintaining perceptual contrast between the two vowels. This possibility is addressed in Experiment 2.

## 5. Experiment 2: audiovisual perception of lip rounding

As demonstrated in Experiment 1, the production of the /ɑ/-/ɔ/ contrast by Michigan speakers is variable, such that some speakers produce the contrast with fewer articulatory gestures than may be expected. These patterns result in a weaker acoustic contrast between the two vowels, which raises the possibility of listeners misperceiving the vowels and failing to acquire the contrast. One question that remains, however, is whether the contrast may be maintained by means other than formant quality. A wealth of evidence supports the notion that listeners are sensitive to visual and other non-auditory speech cues and that such cues may aid listeners in perceiving contrasts. Traunmüller and Öhrström (2007a), for instance, argue that the Swedish /i/-/y/ contrast is perceived in large part visually, rather than auditorily. On the other hand, when visual speech cues are variable, perceptual strength is diminished, as demonstrated for the English dental fricative by McGuire and Babel (2012). They argue that /θ/ is less perceptually stable than /f/ because /θ/ exhibits variation between dental and interdental articulations. In the case of /ɑ/ and /ɔ/, two of the articulatory patterns observed in Experiment 1 (Patterns A and B) maintain a visible rounding distinction between the two vowels, while the third pattern (C) contrasts the vowels through a less-visible distinction in tongue position. As a result, although both Patterns B and C are acoustically weaker than Pattern A, it may be the case that the /ɑ/-/ɔ/ contrast is more easily perceived when produced via Pattern B, which maintains lip rounding, than when produced with Pattern C, which does not. This hypothesis was tested through an audiovisual perception experiment.

### 5.1. Methods and materials

#### 5.1.1. Participants

Thirteen native Michiganders (nine men, four women) were recruited for the perception experiment, but four were excluded from analysis. One participant was excluded because of exceptionally slow response times; the mean response time for this participant was more than twice as long as for any other participant. Two participants were excluded because the demographic questionnaire revealed that they were not raised in Metro Detroit; one participant had been raised in West Michigan, and the other participant had been raised in Northern Ohio. Finally, one participant was excluded because he stated in the post-experiment survey that several of the stimuli rhymed with both response choices (and that both response choices rhymed with each other), making his responses an unreliable indicator of whether he perceived a stimulus as containing /ɑ/ or /ɔ/. For example, this participant stated that he considers *goth* /ɡɑθ/ and *cloth* /klɔθ/ to be rhyming words; whether this indicates that a merger of /ɑ/ and /ɔ/ is already underway for some Michigan speakers, or whether this is simply a lexical difference is a point to address in future research. The remaining nine participants comprised six men and three women, with an age range of 21–41 years old (mean = 26.2 years). All but two of the participants resided in Michigan at the time of the study; the other two participants resided in Washington, DC.

#### 5.1.2. Stimuli

Stimuli for the perception experiment come from a list of 100 monosyllabic nonce words, which were created from the onsets /d, z, θ, ð, st, sk, sl, pl, skl/ and the codas /t, d, θ, ð, k, kt, ks, ts, dz/. These segments were combined with six vowels, comprising three round-unround pairs: the target pair /ɑ ɔ/ and the filler pairs /i u/ and /e o/. These particular onsets and codas were chosen from among the full set of phonotactically permissible English onsets and codas based on two criteria. First, in order to keep the stimulus list balanced across vowels, an onset/coda combination was rejected when the insertion of any of the target or filler vowels would form a real word of English. For example, the onset/coda combination [z_k] was rejected because *Zeke* [zik] is an existing word of English, even though [zuk], [zek], [zok], [zɑk], and [zɔk] are all viable nonce words.^7^ The second condition was that each nonce word was required to rhyme with at least one real monosyllabic word of English, which was used as a response choice in the identification task, described in section 5.1.3.

The stimuli were recorded in a sound-attenuated booth at Georgetown University. Stimuli were recorded using an AKG P420 condenser microphone set to a cardioid polar pattern and an Olympus LS-100 portable solid state recorder at a 44.1 kHz sample rate and 16 bit sample depth. The microphone was mounted to a microphone stand placed near the speaker, but outside the video frame. Video was simultaneously recorded at 30 fps with a Canon X10 camcorder in 1, 920 × 1, 080 resolution. The nonce words were presented to the talker as a list in ARPABET transcription, with nonce words grouped by the vowel they contained, in order to maintain consistency between productions. The stimuli were both recorded and presented embedded in the carrier phrase “say _____ again.” The talker was trained to read the transcriptions and instructed to produce the target vowels as distinctly as possible and to read the stimuli at a consistent pace.

In order to test the relative perceptibility of articulatory patterns B and C, a talker was chosen who produces a relatively weak acoustic contrast between /ɑ/ and /ɔ/, yet who produces /ɔ/ with visible lip rounding (i.e., Pattern B). The talker chosen to produce the stimuli was Speaker 4 from Experiment 1. Because this speaker produces /ɑ/ and /ɔ/ with distinct lip configurations, it is possible to simulate the condition wherein speakers produce /ɔ/ with no discernable lip rounding (Patten C) by splicing video of unround /ɑ/ onto tokens containing auditory /ɔ/. In this way, the visual perceptibility of both articulatory patterns can be tested while controlling for auditory perceptibility and avoiding the effects of interspeaker differences that would arise if stimuli from two talkers were used to compare the two conditions, rather than cross-splicing video from a single speaker. Likewise, it is possible to create visually round variants of tokens containing auditory /ɑ/; while this pattern was not observed in Experiment 1, and is not expected to occur in natural production given to the direction of change in the NCVS, it was included as a condition in the perception experiment. Thus, the audio recording for each target stimulus was paired with one of two video recordings: the original, congruous video, and video which was incongruous in lip rounding. That is, each item containing auditory /ɑ/ or /ɔ/ was paired with video of both unround /ɑ/ and round /ɔ/. Incongruous stimuli were created such that the midpoints of the vowels in the auditory and visual components of the stimulus were aligned. Stimuli were scaled to 70 dB mean RMS amplitude and pink noise was added to the stimuli at a signal to noise ratio of 12 dB.

In order to verify that the target stimuli did, in fact, exhibit a visible contrast in lip rounding, lip measurements were taken at the point of maximum constriction for each token containing /ɑ/ or /ɔ/. Horizontal lip spread and vertical lip openness were measured in the same manner as described for Experiment 1. A two sample *t*-test was conducted for each measure. It was found that both vowels differed significantly from one another in both vertical lip openness (*p* < 0.005) and horizontal lip spread (*p* < 0.001). In order to test the degree of acoustic similarity between the target stimuli, formant measurements for each target nonce word were taken following the methods described for Experiment 1. Formant measurements were normalized using the Labov *ANAE* normalization method (Labov et al., 2006) and a Pillai score was calculated for the two vowels. The resulting Pillai score was 0.47, indicating a higher degree of overlap between the two vowels than observed in the production experiment. Thus, the stimuli used for this experiment accurately represent the desired experimental condition: productions of /ɑ/ and /ɔ/ that are acoustically similar, yet exhibit visible differences in lip rounding.

#### 5.1.3. Procedure

The perception experiment took place in a sound-attenuated booth at the University of Michigan but, as noted above, two participants completed the experiment at Georgetown University. Identical methods and equipment were used in both study locations. Participants were seated in front of a computer monitor placed approximately 1 m away from the participant, with stimuli displayed at eye level. Audio was presented through Sennheiser PC 363D headphones. Stimuli were presented in pseudorandom order, such that no two target stimuli appeared in sequential order. The presentation order was unique to each participant. After each stimulus was presented, participants identified the perceived vowel by selecting a rhyming word of English from one of two choices presented on screen: one word containing the same vowel as the auditory component of the stimulus, and one word containing the vowel with opposing roundedness. For example, the choices for stimuli containing auditory [θɑk] or [θɔk] were “hock” and “hawk,” while the choices for stimuli containing auditory [θek] or [θok] were “fake” and “folk.” The rhyming task was chosen due to ambiguities in English orthography for the vowels /ɑ/ and /ɔ/. Both vowels are represented by a variety of spellings and exhibit overlap in that both vowels can be represented by 〈o〉 (e.g., *cot* vs. *dog*) and 〈a〉 (*palm* vs. *ball*),^8^ which would preclude the use of consistent labels in a traditional identification task.

Participants selected their response by pressing a colored button on a Cedrus RB-30 response pad, which recorded both their response and their reaction time (calculated using the response pad's internal timer). To verify participant attention to the visual stimuli, participants were periodically shown a filler stimulus in which the speakers lips had been digitally colored red, green, orange, or purple. Participants were given two choices and asked to identify the speaker's lip color. An attention question was answered incorrectly only once by a single participant, who was the participant previously excluded from analysis because he did not reliably distinguish between the response choices. In addition, the perception task was video recorded using a camera mounted above the computer display. Trials for which it was clear that the participant was not looking at the computer screen (e.g., looking down at the keyboard, up at the ceiling, off to the side, etc.) would be flagged and excluded from analysis. However, no trials were ultimately excluded in this manner.

### 5.2. Results

**Figure 11:**
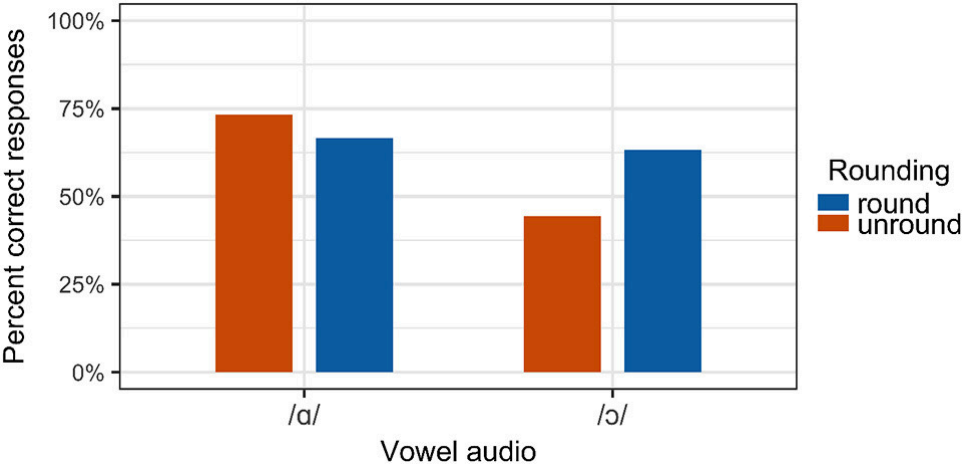
Perception results for all participants.

It was predicted that the rate of correct perception (that is, a response matching the auditory component of the stimulus) would be higher for congruous stimuli than for incongruous stimuli.^9^ For the stimuli containing auditory /ɔ/, this prediction is largely borne out, as shown in Figure 11. For auditory /ɑ/ stimuli, the overall rate of perception of a stimulus as /ɑ/ was similar in both congruous and incongruous conditions.

Results were analyzed using mixed-effects logistic regression using lme4 in R (Bates et al., 2015; R Core Team, 2016), with fixed effects of auditory vowel quality and visual congruity, and random effects of subject and item. The results of the model are presented in Table 2. A significant effect of visual congruity is observed, such that /ɔ/ is “misperceived” as /ɑ/ when produced without lip rounding. This finding supports the hypothesis that articulatory configurations in which /ɔ/ is produced with unround lips are perceptually weaker than those in which /ɔ/ is produced with rounding, even when these configurations result in acoustically identical output.

**Table 2:** Mixed effects logistic regression model for responses in Experiment 2.

	**Estimate**	**Std. Error**	***z*-value**	**Pr(>|z|)**
Intercept	0.5128	0.3310	1.549	0.1213^*^
Vowel (cot)	0.8179	0.3255	2.513	0.0120^*^
Congruity (mismatch)	−0.6465	0.3239	−1.996	0.0459^*^

* *p < 0.05*.

**Figure 12:**
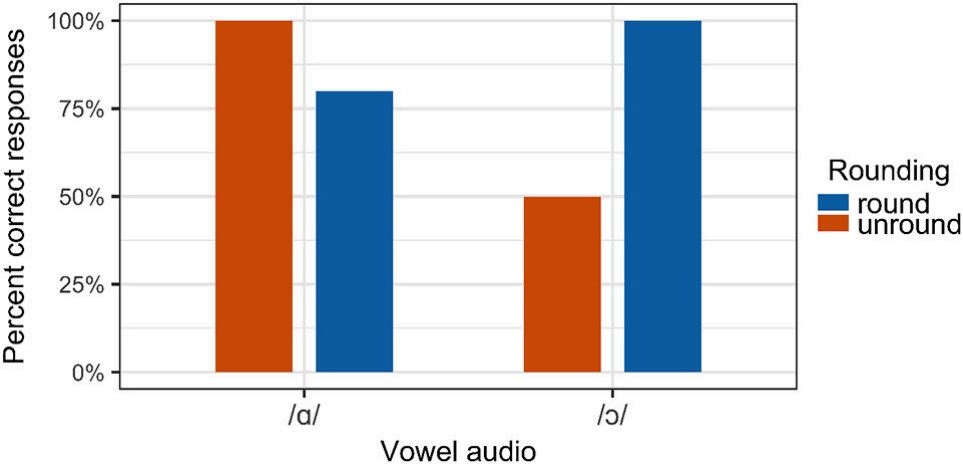
Perception results for Participant 2.

**Figure 13:**
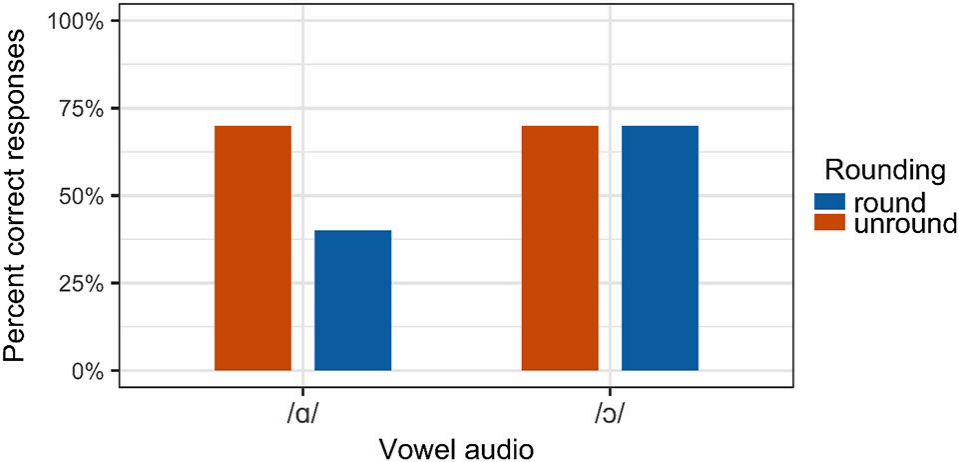
Perception results for Participant 8.

Notably, however, a range of individual variation was observed in the degree and direction to which misperception occurred. For some participants, a loss of rounding on /ɔ/ has a stronger effect than the addition of rounding to /ɑ/, while other participants correctly perceive /ɔ/ even when it is produced with unround lips. For instance, individual results for Participant 2 are presented in Figure 12. This participant correctly perceived both /ɑ/ and /ɔ/ stimuli in 100% of congruous trials. However, when /ɔ/ was presented incongruously with unround lips, perception of the vowel as /ɔ/ was at chance. When /ɑ/ was presented with round lips, it was perceived as /ɔ/ in 20% of trials. In contrast, Participant 8 perceived /ɔ/ stimuli as /ɔ/ at the same rate in both congruous and incongruous conditions, as shown in Figure 13, indicating that the removal of visible lip rounding from /ɔ/ had no effect on this participant's ability to correctly perceive the vowel. Yet the addition of lip rounding to /ɑ/ caused this participant to perceive such stimuli as /ɔ/.

### 5.3. Discussion

In this experiment, a significant overall effect of visual congruity was observed, such that /ɔ/ was more likely to be perceived as /ɑ/ when presented without visible lip rounding cues. This result suggests that the type of articulatory variability observed in Pattern C in Experiment 1 is perceptually weaker than Pattern B, due to its lack of a reliable visual cue to vowel roundedness. The perceptual weakness of this configuration may have several implications for language variation and change, which are discussed in section 6. In addition, a range of individual differences in perception were observed, suggesting avenues for future research.

## 6. General discussion

The experiments presented in this study provide evidence that speakers exhibiting the NCVS differ in the articulatory patterns used to maintain the contrast between /ɑ/ and /ɔ/ and that these articulatory patterns differ in their perceptibility. While some speakers contrast these vowels through a difference in tongue position, others maintain the contrast with a difference in lip rounding or with differences in both tongue position and lip rounding. These strategies differ in their acoustic output, such that speakers who maintain a difference between the vowels along only one articulatory dimension exhibit a relatively weaker acoustic contrast than speakers who produce differences in both tongue position and lip rounding. While the strength of the acoustic contrast is similar for both single-articulator strategies, the results of the perceptual study suggest that these strategies are not equal in their perceptibility. When participants were presented with tokens of /ɔ/ produced with unround lips, they were significantly more likely to perceive the vowel as /ɑ/ than when it was produced with visibly round lips. This result suggests that articulatory configurations in which /ɔ/ is produced with unround lips are more likely to be (mis)perceived as /ɑ/ than tokens of /ɔ/ produced with rounding.

We interpret these findings to indicate that visual speech perception cues may influence patterns of sound change and variation in at least two ways. First, these findings suggest that visual cues may play a role in shaping phonological systems through misperception-based sound change. Listener-based theories of change (Ohala, 1993; Blevins, 2004) posit that the primary source of sound change is in the imperfect transmission of the phonetic signal across a noisy channel, but previous research in this area has generally been limited to auditory perception. For instance, Ohala (1981) suggests that the acoustic similarity of /θ/ and /f/ is a source of misperception of these two sounds. This suggestion is supported by the findings of Miller and Nicely (1955), who show that these sounds are frequently confused in auditory perception tasks. On a purely acoustic/auditory account, one would predict /θ/ > /f/ sound changes to be symmetrical, such that /θ/ > /f/ and /f/ > /θ/ changes would occur with equal frequency. However, /θ/ and /f/ differ crucially in that their articulations are visibly distinct. McGuire and Babel (2012) show that this visual distinction facilitates listener identification of the two fricatives, such that identification is more accurate in an audiovisual condition than in audio-only or video-only conditions, but that articulatory variability for /θ/ makes this sound perceptually weaker than /f/.

In the case of /ɑ/ and /ɔ/ presented here, perceptual weakness may similarly arise due to variability in the rounding of /ɔ/. In speech communities where both /ɑ/ and /ɔ/ are produced with unround lips or where /ɔ/ is only sometimes produced with rounding, the contrast between /ɑ/ and /ɔ/ will be perceptually weaker than in communities where /ɔ/ is always produced with rounding and thus visually contrastive with /ɑ/. The findings in this study predict that such a situation may over time lead language learners to misperceive /ɔ/ as /ɑ/, with the potential for merger of these vowels. While it is generally recognized to be difficult or impossible to predict future sound changes,^10^ continued work on the articulatory patterns underlying the NCVS may shed light on ongoing change in the region. Recent research has shown that, among younger speakers, the NCVS is in decline and reversing in many cities (Dinkin, 2009; McCarthy, 2010; Friedman, 2014; Driscoll and Lape, 2015). For speakers from Lansing, Michigan, Wagner et al. (2016) find that /ɑ/ is receding from its previously fronted position and returning to a more canonical low back position. In addition, they find that some speakers in their sample exhibit a merger of /ɑ/ and /ɔ/. One might predict that if speakers produce /ɔ/ with unround lips and /ɑ/ begins to back, the similarity of these vowels in both the auditory and visual domains will make them susceptible to merger.

On the other hand, in cases where pressure to maintain a phonological contrast is high, visibly distinct articulatory variants may be preferred, such that fronted back vowels will tend to retain their rounding. Following the model of Lindblom et al. (1995), language users who are exposed to both round and unround productions of /ɔ/ will evaluate both of these variants, selecting one or the other depending on articulatory, perceptual, and social factors. In contexts where perceptual demands are high, speakers are predicted to prefer round variants of /ɔ/, given that the addition of visible lip rounding enhances the contrast with /ɑ/. This prediction is supported by the findings of Ménard et al. (2016), who show that sighted speakers consider how their speech will be perceived both visually and auditorily, and increase the degree of lip rounding for /u/ in clear speech. The integration of visual speech cues may therefore offer an explanation for the finding of Harrington et al. (2011) that British English /u/ has retained its rounding as it has undergone fronting: in acoustic terms, both [y] and [ɨ] should be viable articulations for fronted /u/, but visibly round [y] is predicted to maintain a stronger contrast with /i/. For patterns of articulatory variation where neither variant has strong visual cues, as in the tense [æ] variants described by De Decker and Nycz (2012) or bunched vs. retroflex variants of /ɹ/, both articulatory variants are predicted to be perceptually equivalent. While additional research is needed to confirm whether this pattern holds more broadly, it presents a potential challenge for theories that consider sounds solely in terms of auditory perceptibility. For instance, Diehl and Kluender (1989) explicitly argue against the notion that vowels are dispersed in the articulatory domain. They correctly observe that vowel systems containing /i u a/ are cross-linguistically preferred, while systems composed of /y ɯ a/ are unattested. They explain that while both vowel systems are equally dispersed in the articulatory domain, only /i u a/ exhibits maximal dispersion in both the articulatory and auditory domains. While their theory of auditory enhancement makes the correct prediction for typical vowel systems, it makes no prediction as to what will happen when a back vowel (such as /u/ or /ɔ/) is fronted as a result of sound change. If phonological contrasts are optimized for both auditory *and* visual perceptibility, however, vowels are predicted to retain their rounding in order to maintain contrasts with the front unround vowels.

One question which remains is why the sort of articulatory variation observed in the production experiment should arise in the first place if unround variants of /ɔ/ are dispreferred on perceptual grounds. McGuire and Babel (2012) raise a similar question with respect to articulatory variability for /θ/ and note that some degree of articulatory variability is to be expected due to coarticulation. Similarly, in the case of /ɔ/, it is possible that listeners fail to detect rounding in certain phonetic contexts. For instance, when /ɔ/ appears next to a labial or rounded segment such as /ʃ/ (as in [ʃɔn] *Sean*) or /p/ (as in [pɔ] *paw*), listeners may attribute the rounding on /ɔ/ to the neighboring consonant rather than to the vowel itself. This likelihood may be increased for low round vowels like /ɔ/, which typically exhibit a smaller degree of rounding than high vowels due to the openness of the jaw (Ladefoged and Maddieson, 1996). Ongoing work on the articulation of the NCVS considers more closely the effects of phonetic environment on the realization of lip rounding for /ɔ/. Another issue is that it is difficult to make generalizations about the frequency of the observed articulatory patterns due to the small number of speakers in the production experiment. Notably, among the participants in this study, there was only one speaker who contrasted /ɔ/ from /ɑ/ through tongue position alone. It is therefore unclear whether this pattern is widespread or whether it is simply an idiosyncracy of this particular speaker. Collection of articulatory data from a larger sample of speakers is underway, and will help to address this question. If the expanded articulatory study finds that producing /ɔ/ with unround lips is rare, it will support the hypothesis that unround variants of /ɔ/ are in fact dispreferred.

Another question to address in future research concerns the observed individual variation in the perception experiment. It was found that the direction in which misperception occurred and the degree to which listeners were influenced by visual incongruity varied on a listener-by-listener basis. For some listeners, a loss of lip rounding on /ɔ/ resulted in misperception as /ɑ/, while other listeners correctly perceived /ɔ/ regardless of whether it was presented with lip rounding. For some such speakers, addition of rounding to /ɑ/ resulted in misperception as /ɔ/. One possibility is that some listeners were simply less sensitive to the visual component of the stimuli than others, either because of the particular talker used to produce the stimuli or because the listener relies more on auditory cues in speech perception more generally. Traunmüller and Öhrström (2007a) find that some listeners rely more heavily on auditory cues than on visual cues when perceiving rounding contrasts in Swedish, while Kricos (1996) shows intertalker differences in listeners' ability to lipread speech. While the talker for the present perception study was selected for her specific acoustic and articulatory realizations of /ɔ/ and /ɑ/, ongoing work incorporates stimuli from multiple talkers. Another possibility is that a listener's reliance on visual lip rounding cues for /ɔ/ depends on their own use of lip rounding in speech production. It may be the case that listeners who produce /ɔ/ with unround lips are influenced less by incongruous audiovisual stimuli because they do not rely on lip rounding to produce the /ɑ/-/ɔ/ contrast in their own speech.

## 7. Conclusion

The results of the present study suggest that audiovisual speech perception can influence patterns of sound change, with implications for the development of phonological systems. First, our findings suggest that visual cues can shape phonological systems through misperception-based change, by making visibly distinct articulatory patterns less susceptible to misperception. Moreover, this finding suggests that phonological systems may be optimized not only for auditory and for visual perceptibility. Thus, the current study shows that a comprehensive theory of language variation and sound change must consider how speech is conveyed across a variety of perceptual modalities.

## Ethics statement

This study was carried out in accordance with the recommendations of the Georgetown University Social and Behavioral Sciences Institutional Review Board (IRB-C) with written informed consent from all subjects. All subjects gave written informed consent in accordance with the Declaration of Helsinki. The protocol was approved by the Georgetown University IRB-C.

## Author contributions

The articulatory experiment was conceived and carried out by JH. The perception experiment was developed from JH's project for a seminar where YD was the instructor. JH was responsible for setup and data collection for both experiments. Both authors contributed to the formulation of research questions, experimental design, data analysis, and writeup of the results.

### Conflict of interest statement

The authors declare that the research was conducted in the absence of any commercial or financial relationships that could be construed as a potential conflict of interest. The reviewer, SH, and handling Editor declared their shared affiliation.

## Footnotes

^1^A notable exception is a study by Plichta (2004), who argues on the basis of nasal/oral airflow data that the acoustic raising of /æ/ in the NCVS may be the result of nasalization that occurs even in non-nasal environments, such that the [æ] in *dad* is nearly as nasalized as that in *man*.

^2^For the purposes of this study, Metro Detroit was defined as the Detroit-Warren-Flint Combined Statistical Area. One speaker was raised just outside this area in neighboring Jackson County, Michigan.

^3^The extent to which participants' speech production may have changed as a result of having lived outside of Metro Detroit is not considered.

^4^When necessary, video frames were rotated during extraction to account for camera tilt. The degree of required rotation varied between 2° and 5.5°.

^5^The implementation of gss::ssanova used here is based on code provided by Fruehwald (Unpublished Manuscript) and Mielke (2013).

^6^Based on plots presented by Nycz and Hall-Lew (2014) and Di Canio (2013).

^7^Due to an oversight, *zoot* [zut] was included in the stimulus list, but this word is judged to be marginal as it occurs only as part of the phrase *zoot suit*.

^8^Like most varieties of American English, the dialect of the Inland North exhibits the lot-palm merger, such that *palm* is pronounced with [ɑ].

^9^Responses matching the auditory component of the stimulus are considered “correct” because the incongruous stimuli containing auditory /ɔ/ are intended to simulate articulatory Pattern C, in which /ɔ/ is produced without visible lip rounding. Thus, if a participant perceives one such stimulus as /ɔ/, it is considered to be a correct perception of that vowel as it would be produced by a speaker of Pattern C.

^10^Weinreich et al. (1968, p. 99), for instance, write: “In its strong form, the theory [of language change] would predict, from a description of a language state at some moment in time, the course of development which that language would undergo within a specified interval. Few practicing historians of language would be rash enough to claim that such a theory is possible.”
